# Retraction: miR-26a-5p mediates TLR signaling pathway by targeting CTGF in LPS induced alveolar macrophage

**DOI:** 10.1042/BSR-2019-2598_RET

**Published:** 2023-02-27

**Keywords:** connective tissue growth factor, miR-26a-5p, severe pneumonia, toll-like receptor signaling pathway

This Retraction follows an Expression of Concern relating to this article previously published by Portland Press. This article is being retracted from *Bioscience Reports* at the request of the Editor-in-Chief and the Editorial Board following receipt of a notification from a reader, alerting the Editorial Board to concerns over the flow-cytometry data, some of which doesn't appear to be feasible. The western blot original data also contains features shared amongst other original western blots presented by other author groups. The authors have been contacted with regards to the retraction and have not responded to the Journal's queries and the concerns raised. Given the extent of the issues raised, the Editorial Board stand by the decision to retract the article.

